# A study and benchmark of DNcon: a method for protein residue-residue contact prediction using deep networks

**DOI:** 10.1186/1471-2105-14-S14-S12

**Published:** 2013-10-09

**Authors:** Jesse Eickholt, Jianlin Cheng

**Affiliations:** 1 Department of Computer Science, Central Michigan University, Mt. Pleasant, MI 48859, USA; 2 Department of Computer Science, University of Missouri, Columbia, MO 65211, USA; 3 Informatics Institute, University of Missouri, Columbia, MO 65211, USA; 4 C. Bond Life Science Center, University of Missouri, Columbia, MO 65211, USA

**Keywords:** protein residue-residue contact, contact prediction, deep learning, deep networks

## Abstract

**Background:**

In recent years, the use and importance of predicted protein residue-residue contacts has grown considerably with demonstrated applications such as drug design, protein tertiary structure prediction and model quality assessment. Nevertheless, reported accuracies in the range of 25-35% stubbornly remain the norm for sequence based, long range contact predictions on hard targets. This is in spite of a prolonged effort on behalf of the community to improve the performance of residue-residue contact prediction. A thorough study of the quality of current residue-residue contact predictions and the evaluation metrics used as well as an analysis of current methods is needed to stimulate further advancement in contact prediction and its application. Such a study will better explain the quality and nature of residue-residue contact predictions generated by current methods and as a result lead to better use of this contact information.

**Results:**

We evaluated several sequence based residue-residue contact predictors that participated in the tenth Critical Assessment of protein Structure Prediction (CASP) experiment. The evaluation was performed using standard assessment techniques such as those used by the official CASP assessors as well as two novel evaluation metrics (i.e., cluster accuracy and cluster count). An in-depth analysis revealed that while most residue-residue contact predictions generated are not accurate at the residue level, there is quite a strong contact signal present when allowing for less than residue level precision. Our residue-residue contact predictor, DNcon, performed particularly well achieving an accuracy of 66% for the top L/10 long range contacts when evaluated in a neighbourhood of size 2. The coverage of residue-residue contact areas was also greater with DNcon when compared to other methods. We also provide an analysis of DNcon with respect to its underlying architecture and features used for classification.

**Conclusions:**

Our novel evaluation metrics demonstrate that current residue-residue contact predictions do contain a strong contact signal and are of better quality than standard evaluation metrics indicate. Our method, DNcon, is a robust, state-of-the-art residue-residue sequence based contact predictor and excelled under a number of evaluation schemes. It is available as a web service at http://iris.rnet.missouri.edu/dncon/.

## Background

Protein residue-residue contact prediction is a long standing and largely unsolved problem in Structural Bioinformatics with some of the earliest methods being developed nearly 20 years ago [1]. Since then, the relevance and importance of the problem has grown due to the usefulness of predicted contact information. To date demonstrated uses of predicted protein residue-residue contact information include rational drug design [2], protein model ranking and quality assessment [3,4] and protein tertiary structure prediction [5].

Historically, the prediction of protein residue-residue contacts has been tied to the challenging problem of protein tertiary structure prediction. The use of residue-residue contacts allows the protein modelling problem to be reformulated as a classification task. In this reformulated setting, it is only necessary to determine which residues are in contact and then use the contacting residue pairs to generate protein structures from inferred distance restraints. In practice, however, it has proven difficult to generate protein structures from predicted contacts due to the relatively low accuracy of most residue-residue contact prediction methods. Accuracies for the top, long range predicted residue-residue contacts are often in the range of 25-35% [6,7]. Residue-residue contact predictions of this quality are too noisy for current reconstruction tools [8].

A majority of the sequence based contact prediction approaches developed make use of machine learning. These methods attempt to learn a mathematical function which maps extracted features to a real valued number representing the predicted class (i.e., contact or non-contact). The features used as input to the function are values representing different characterizations of the protein's sequence which are believed to be relevant for the classification task at hand. A number of particular machine learning approaches have been used including neural networks [9-13], support vector machine [14,15], deep learning [16,17] and random forest [18].

Some of the first methods developed attempted to identify residue-residue contacts using evolutionary information contained in multiple sequence alignments (MSAs). This was done by locating residue-residue pairs which coevolved [1,19,20]. While fast and conceptually attractive, these methods typically did not perform very well due to difficulties in distinguishing true, coevolving residue pairs from transient relationships (e.g., if residue paris (i,j) and (j,k) are in contact and coevolve, it would appear that residues i and k also coevolved). Newer techniques such as those used by EVFold [21]and PSICOV [22] are better able to distinguish true residue-residue contacts and generate rather accurate predictions provided that a large, diverse multiple sequence alignment can be formed for a query protein. These approaches have lead to a breakthrough in performance and reached levels were reconstruction from predicted contacts is possible. The drawback, however, is often these approaches are not applicable. This is because they require the construction of a large and diverse multiple sequence alignment which is not always possible, particularly in the case of hard protein modelling targets.

Hard protein modelling targets are targets for which structural information (i.e., a protein template) does not exist or is not detectable by a sequence based search. These types of targets are the most difficult to model and evaluate and are precisely the types of targets which could most benefit from residue-residue contact data. As a result, a residue-residue contact predictor is typically evaluated based on its performance on hard, or free modelling (FM), targets. Unfortunately, the reported performance of predicted residue-residue contacts on these types of hard targets is typically low, making it difficult to understand how predictions of this quality can be of practical use. Thus, there is a need to further analyze the quality and value of predicted residue-residue contacts for hard targets and the methods used.

Here we present a broad analysis of residue-residue contact predictors which participated in the tenth round of the Critical Assessment of protein Structure Predictions (CASP). In the analysis, we used both standard evaluation metrics such as those used by the CASP assessors as well novel, non standard metrics (e.g., evaluation by neighbourhood, cluster accuracy and cluster count). The additional evaluation metrics we use show a strong contact signal present in residue-residue contact predictions and help clarify a seeming contradiction between the usefulness of contact predictions and reported accuracies.

Our method, DNcon, performed well under all of the evaluation metrics employed and in particular, DNcon was among the best contact predictors in terms of accuracy, accuracy in a neighbourhood and cluster count. Given its performance in CASP10, we also present a study of the underlying architecture and features used in DNcon. We found that DNcon is particularly robust with respect to the number of layers used and number of nodes per layer in its underlying boosted, deep network architecture.

## Methods

Every two years the current state-of-the-art in protein structure prediction methods is evaluated in the CASP experiment. Over a period of several months, protein sequences which do not have known experimentally determined structures are sent out for prediction. Each participant in the residue-residue contact prediction category had approximately three days to score residue-residue pairs for contact and send their predictions back to the Prediction Center. The Prediction Center collects the results for each participant and makes them available to the public after the prediction season. In this assessment, the primary source of residue-residue contact predictions for the methods considered was the official CASP10 website [23].

In total more than 25 methods registered in the protein residue-residue prediction category. We limited our evaluation to those methods which made predictions for a vast majority of the CASP10 targets and were, to the best of our knowledge, *ab-initio *in nature (i.e., methods which did not directly use template information in the contact prediction process). These methods were chosen since results obtained would generalize to the hard (or free) modelling targets as they do not directly make use of template information. The methods selected included two approaches based on random forests (groups 257 and 396), an approach using a support vector machine (group 081), an approach using recursive neural networks (group 125), an approach using deep learning (group 305) and our approach DNcon (group 222). Descriptions for these methods as well as all of the methods that participated in CASP10 can be found in the abstracts on the official CASP10 website [23].

DNcon is a sequence based, ab-initio residue-residue contact predictor built upon a combination of boosting and deep networks (DNs). Conceptually, each DN in the boosted ensemble is similar to a standard two layer neural network but has many more layer and trained in a step wise, semi-supervised fashion. The input features to each deep network stem primarily from two fixed width windows centred on the residue-residue pair to be classified. From the residues contained in these windows a number of features are encoded such as predicted secondary structure and solvent accessibility, primary sequence, sequence profile from a position specific scoring matrix (PSSM) and various statistical characterizations of the residues (e.g., Atchley factors [24]). Some global information about the protein such as sequence length and content was also used. More specifically, secondary structure and solvent accessibility values were predicted using SSpro and ACCpro from the SCRATCH suite [25]. To obtain the values for the PSSM, PSI-BLAST [26] was run for three iterations against a non-redundant version of the nr sequence database filtered at 90%. For full details on feature generation and the construction and training of DNcon, see Eickholt and Cheng [17].

The primary dataset used in this benchmark is the CASP10 dataset. It consists of 96 protein targets used in CASP10 whose experimentally determined structures were available on the official CASP website [23] at the time of this study. We also considered a subset of these 96 proteins which we term the HARD CASP10 targets. These are 13 protein targets which contained at least one protein domain which was preliminarily classified as free modelling or free modelling/template based modelling according to the CASP10 website [23]. For the additional analysis we performed on DNcon with respect to architecture and feature selection, we used 111 valid protein targets from CASP9 as the evaluation dataset. Protein sequences and target structures were obtained from the official CASP9 website [27]. Training was performed using the DNCON_TRAIN dataset, a collection of 1230 proteins used to train DNcon [17].

Two amino acid residues are said to be in contact if the distance between their respective Cβ atoms (Cα for glycine) is less than 8 Angstrom. This is a standard definition of protein residue-residue contact and has been used in a number of previous studies and official CASP assessments [6,7,9,18]. Residue-residue contacts are further classified as short, medium or long range contacts based on their separation in the protein sequence. Short range contacts are defined as residue-residue contacts with a sequence separation of 6 to 12 residues, medium range contacts have a sequence separation from 12 to 24 residues and long range residue-residue contacts are those separated by 24 or more residues in sequence. This additional differentiation of residue-residue contacts is useful as the shorter range contacts tend to be easier to predict and less useful while longer range contacts present more of a challenge and contain more information about the overall conformation of a protein.

Given the difficulty in predicting protein residue-residue contacts, methods are often evaluated by considering the accuracy of the top L/n predicted contacts where L is the length of evaluation target and n is a small integer (e.g., 1, 5 or 10). In this setting accuracy is defined as the percent of residue-residue pairs considered that are true residue-residue contacts divided by the number of predictions considered (e.g., if the top 20 residue-residue contact predictions for a protein are considered and 10 of these pairs are in contact in the experimentally determined structure, then the accuracy for this protein would be 0.50). Estimates for the standard error (SE) were obtained using the sample mean and sample variance of the per target accuracies over the dataset considered.

Along with the standard evaluation metric of residue-residue accuracy, we also used a number of additional evaluation metrics including two novel metrics which we developed. The first additional metric we term accuracy in a neighbourhood and it calculates the accuracy of predictions when allowing for less than residue level precision. In this setting, a residue-residue pair is counted as correct if there is a true contacting pair within +/- δ, for small values of δ (e.g., 1 or 2). The second and third additional evaluation metrics combine a neighbourhood evaluation with clustering. Here the selected contact predictions (e.g., top L/5) are first filtered using a greedy clustering approach (see Results section for full details). The clusters can then be checked for accuracy and separation (i.e., cluster accuracy and cluster count). The rationale behind these metrics is to study the distribution of the predicted residue-residue pairs among the top scoring predictions and ensure that the predictions are not clustered around a few interactions.

Finally, we mention that the evaluation unit in this study is the full protein and we evaluate performance irrespective of any underlying domain architecture. This is different from the approach used by the official CASP assessors which typically evaluate predictions on a per domain basis. Our assessment over the full protein and our development and use of additional evaluation metrics (i.e., by neighbourhood and clustering) is meant to complement the evaluation provided by official CASP assessors. The evaluation provided by the CASP assessors is finer in nature and at the residue level (i.e., a prediction is counted as correct if it identifies a contacting residue pair present in the experimentally determined structure). Our evaluation is at a courser level of resolution and characterizes how well predicted residue contacts describe areas of interaction in the protein chain. A summary of the official CASP10 residue-residue contact prediction results is available at: http://www.predictioncenter.org/casp10/rr_summary_results.cgi.

## Results

### Performance in CASP10

Protein residue-residue contact predictors are best evaluated on hard protein modelling targets as these are the types of targets for which predicted contact information could be of most use. Table 1 reports the accuracy of DNcon's top L/10, L/5 and L medium and long range predictions along with several other sequence based predictors on 13 hard CASP10 targets using standard evaluation metrics. The results of this evaluation indicate that the methods can be grouped into three sets when considering the top L/10 long range predictions. The best set of methods achieves accuracies in the range of 0.21-26 followed by sets with accuracies in the ranges of 0.12-15 and 0.08-0.09. The distinction between these three groups is visible as well when considering the top L/5 long range contacts but breaks down when considering the top L long range or any set of medium range contact predictions. For both medium and long range contact predictions on these targets, DNcon is consistently among the best predictors.

**Table 1:** Performance on HARD CASP10 targets

	Acc. Top L/10 (SE)		Acc. Top L/5 (SE)		Acc. Top L (SE)	
			
Method (GroupID)	Long	Medium	Long	Medium	Long	Medium
IBGteam [DL] (305)	0.263 (0.066)	0.356(0.085)	0.208 (0.050)	0.292(0.063)	0.117 (0.024)	0.180(0.027)
DNcon (222)	0.244 (0.039)	0.442(0.072)	0.207 (0.029)	0.346(0.056)	0.128 (0.016)	0.206(0.034)
RandomForest (396)	0.228 (0.063)	0.336(0.066)	0.193 (0.057)	0.283(0.047)	0.122 (0.020)	0.159(0.019)
RandomForest (257)	0.208 (0.067)	0.336(0.066)	0.195 (0.091)	0.283(0.047)	0.119 (0.091)	0.159(0.019)
RaptorX-Roll (358)	0.146 (0.034)	0.412(0.076)	0.164 (0.028)	0.344(0.058)	0.105 (0.026)	0.271(0.041)
PLCT (332)	0.116 (0.027)	0.329(0.070)	0.095(0.016)	0.275(0.050)	0.073(0.008)	0.173(0.024)
SVM (81)	0.087 (0.03)	0.253(0.061)	0.090 (0.03)	0.243(0.051)	0.069 (0.020)	0.162(0.023)
1d-rec. NN (125)	0.075 (0.022)	0.338(0.076)	0.067 (0.017)	0.290(0.055)	0.046 (0.009)	0.183(0.031)

Accuracies for the top L/10, L/5 and L medium and long range contact predictions for 13 hard targets. L is the length of the protein. Estimates for standard error are provided in parenthesises.

The drawback to evaluating residue-residue contact predictors on hard targets is that the evaluation sets are often small. This is due to the fact that most hard modelling targets are proteins related to new folds and experimentally determined structures for new folds are not as common. To improve the robustness of our assessment, we extended our evaluation to 96 CASP10 targets. Since the methods considered in our benchmark do not make use of template information in the prediction process, this is still a fair assessment and no method has an undue advantage. The results of this extended evaluation are presented in Table 2. As before, the methods can be roughly divided into three sets. The top performing set has accuracies for the top L/10 long range predictions in the range of 0.33-0.36 followed by two methods which achieved accuracies of 0.22-25 and another method with an accuracy of 0.14. Here again, DNcon performed well and among the best methods for both long and medium range contact predictions.

**Table 2:** Performance on CASP10 targets

	Acc. Top L/10 (SE)		Acc. Top L/5 (SE)		Acc. Top L (SE)	
			
Method (GroupID)	Long	Medium	Long	Medium	Long	Medium
RandomForest (396)	0.356 (0.030)	0.455(0.027)	0.314 (0.026)	0.380(0.024)	0.175 (0.013)	0.201(0.013)
DNcon (222)	0.354 (0.027)	0.457(0.027)	0.304 (0.022)	0.381(0.022)	0.176 (0.012)	0.218(0.012)
IBGteam [DL] (305)	0.352 (0.029)	0.422(0.027)	0.298 (0.025)	0.355(0.023)	0.161 (0.013)	0.197(0.011)
RandomForest (257)	0.347 (0.030)	0.455(0.027)	0.298 (0.025)	0.380(0.024)	0.172 (0.013)	0.201(0.013)
RaptorX-Roll (358)	0.331 (0.026)	0.469(0.022)	0.287 (0.044)	0.401(0.020)	0.183 (0.013)	0.313(0.013)
1d-rec. NN (125)	0.252 (0.028)	0.391(0.029)	0.209 (0.032)	0.329(0.025)	0.110 (0.011)	0.189(0.013)
SVM (81)	0.216 (0.023)	0.347(0.025)	0.192 (0.019)	0.297(0.022)	0.120 (0.011)	0.178(0.011)
PLCT (332)	0.142(0.018)	0.369(0.027)	0.123 (0.014)	0.304(0.021)	0.086 (0.008)	0.174(0.011)

Accuracies for the top L/10, L/5 and L medium and long range contact predictions for 96 CASP10 targets. L is the length of the protein. Estimates for standard error are provided in parenthesises.

In this work we also wanted to further examine what is seemingly a contradiction between the usefulness of predicted protein residue-residue contacts and the accuracies achieved by state-of-the-art predictors. The literature contains many documented uses of predicted contacts but this is difficult to understand given the relatively low accuracies, particularly for hard targets. Thus, we evaluated the contact predictions using a neighbourhood as described in the Methods section. This type of evaluation scheme allows for less than residue level precision and counts a predicted contact correct if it is within one or two residues of a true residue-residue contact. Conceptually, predictions of this level of resolution would still be useful for tasks such as model quality assessment and searching the conformational search space as they describe areas of interaction along the protein chain. Tables 3 and 4 show the performance of several predictors using this relaxed evaluation scheme on hard targets and all targets. As shown in Tables 3 and 4, accuracies for the top L/10 long range contacts approach 0.60 and near or surpass 0.70 for the top L/10 medium range contacts. Thus, there is a much stronger contact signal present in the contact predictions than the results from the residue level assessment would indicate and this gives credence to the usability of predicted contact information.

**Table 3:** Performance on HARD CASP10 targets using neighbourhoods (δ)

		Acc. Top L/10(SE)		Acc. Top L/5 (SE)	
			
Method	δ	Long	Medium	Long	Medium
DNcon (222)	1	0.484 (0.067)	0.612 (0.069)	0.438 (0.055)	0.559 (0.057)
IBGteam [DL] (305)	1	0.450 (0.103)	0.546 (0.104)	0.395 (0.086)	0.507 (0.081)
RandomForest (396)	1	0.412 (0.081)	0.505 (0.082)	0.385 (0.067)	0.481 (0.058)
RandomForest (257)	1	0.377 (0.078)	0.505 (0.082)	0.365 (0.066)	0.481 (0.068)
RaptorX-Roll (358)	1	0.349 (0.067)	0.626 (0.084)	0.378 (0.062)	0.591 (0.075)
SVM (81)	1	0.251 (0.050)	0.464 (0.087)	0.243 (0.051)	0.440 (0.073)

DNcon (222)	2	0.619 (0.081)	0.726 (0.058)	0.563 (0.062)	0.674 (0.052)
IBGteam [DL] (305)	2	0.500 (0.112)	0.635 (0.093)	0.427 (0.097)	0.592 (0.074)
RandomForest (396)	2	0.527 (0.079)	0.591 (0.080)	0.486 (0.065)	0.569 (0.058)
RaptorX-Roll (358)	2	0.464 (0.075)	0.692 (0.081)	0.471 (0.068)	0.672 (0.072)
RandomForest (257)	2	0.470 (0.078)	0.591 (0.080)	0.456 (0.066)	0.569 (0.058)
SVM (81)	2	0.409 (0.082)	0.566 (0.086)	0.371 (0.071)	0.537 (0.074)

Accuracies for the top L/10 and L/5 medium and long range contact predictions for 13 hard targets. L is the length of the protein. Estimates for standard error are provided in parenthesises. δ is the size of the neighbourhood.

**Table 4:** Performance on CASP10 targets using neighbourhoods (δ)

		Acc. Top L/10(SE)		Acc. Top L/5 (SE)	
			
Method	δ	Long	Medium	Long	Medium
DNcon (222)	1	0.580 (0.032)	0.674 (0.029)	0.526 (0.029)	0.623 (0.026)
IBGteam [DL] (305)	1	0.555 (0.036)	0.648 (0.030)	0.491 (0.033)	0.609 (0.027)
RandomForest (396)	1	0.534 (0.036)	0.671 (0.030)	0.504 (0.032)	0.628 (0.028)
RaptorX-Roll (358)	1	0.529 (0.031)	0.731 (0.025)	0.490 (0.030)	0.680 (0.024)
RandomForest (257)	1	0.526 (0.036)	0.671 (0.030)	0.484 (0.032)	0.680 (0.024)
SVM (81)	1	0.394 (0.032)	0.598 (0.033)	0.365 (0.028)	0.542 (0.029)

DNcon (222)	2	0.663 (0.032)	0.749 (0.027)	0.615 (0.029)	0.720 (0.024)
RandomForest (396)	2	0.609 (0.035)	0.734 (0.027)	0.577 (0.032)	0.705 (0.026)
IBGteam [DL] (305)	2	0.607 (0.037)	0.729 (0.027)	0.555 (0.034)	0.695 (0.025)
RaptorX-Roll (358)	2	0.606 (0.031)	0.801 (0.023)	0.540 (0.029)	0.764 (0.022)
RandomForest (257)	2	0.597 (0.035)	0.734 (0.027)	0.561 (0.031)	0.723 (0.026)
SVM (81)	2	0.484 (0.034)	0.681 (0.032)	0.451 (0.034)	0.644 (0.029)

Accuracies for the top L/10 and L/5 medium and long range contact predictions for 96CASP10 targets. L is the length of the protein. Estimates for standard error are provided in parenthesises. δ is the size of the neighbourhood.

In order to ensure that the methods were not clustering predicted contacts around a few areas of interaction, we also wanted to consider the distribution or coverage of predicted contacts in the list of the top L/10 or L/5 contacts. This is to say that we wanted to determine how many areas of interactions the contact predictors were identifying. To do this, we first cluster the top L/10 or L/5 contacts for a target in a greedy fashion. For each predicted residue-residue pair considered, we added it to a list of cluster representatives if there was not a residue-residue pair within 8 residues. More specifically, we added residue pair (x,y) only if |x-x_i_| > 8 or |y-y_i_| >8 for all residue pairs (x_i_, y_i_) already in the list of cluster representatives. The list of represented contacts was then counted and evaluated using a neighbourhood of δ = 2. Table 5 presents the results of this study. The cluster count is the number of cluster representatives considered by a method (i.e., the number of areas of interaction considered). In this evaluation, DNcon not only outperforms other methods in terms of the accuracy of the cluster representatives but also in the number of clusters identified. Thus, not only are DNcon's predictions more accurate, they also identify and recover more areas of interaction along the protein chain.

**Table 5:** Performance on CASP10 targets with clustering and neighbourhoods (δ = 2)

	Top L/10 long range		Top L/5 long range	
		
Method	Acc. (SE)	Cluster count	Acc. (SE)	Cluster count
DNcon(222)	0.583(0.030)	666	0.520(0.025)	1018
RaptorX-Roll(358)	0.524(0.030)	596	0.476(0.027)	917
IBGteam [DL] (305)	0.503(0.037)	408	0.391(0.034)	662
RandomForest (396)	0.477(0.035)	577	0.441(0.031)	895
RandomForest(257)	0.455(0.034)	627	0.415(0.030)	907
SVM(81)	0.416(0.034)	596	0.345(0.027)	936

Accuracies for the top L/10, L/5 and L medium and long range contact predictions for 96 CASP10 targets. L is the length of the protein. Estimates for standard error are provided in parenthesises. δ is the size of the neighbourhood. Cluster count is the number of clusters identified by the method.

**Figure 1:**
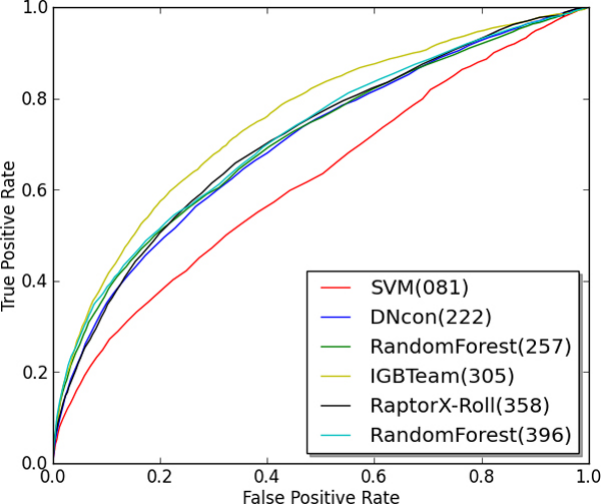
**ROC curve for Top L predictions on CASP10 protein targets**.

**Figure 2:**
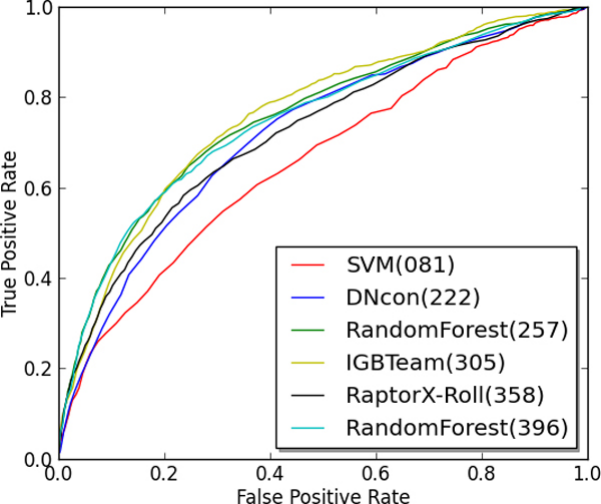
**ROC curve for Top L/5 predictions on CASP10 protein targets**.

The final assessment we performed on the six residue-residue contact prediction methods was an analysis of the ROC curve on the CASP10 benchmark. Figures 1 and Figure 2 show the ROC curve for the methods on top L and L/5 predictions respectively. This was accomplished by collecting the top L (or L/5) ranked predictions for each protein target and then calculating the true positive rate and false positive rate of these contact predictions at a variety of decision thresholds. Tables 6 and 7 show the calculated area under the curve (AUC) for each ROC curve and this value characterizes the overall classification performance of a method across a number of decision thresholds. As Figures 1 and Figure 2 indicate, the deep learning method from group 305 (i.e., IGBteam) performs better across a variety of decision thresholds, particularly when considering the top L1 predictions.

**Table 6:** AUC for the top L predictions on CASP10 targets

Method	AUC
IBGteam [DL] (305)	0.753
RandomForest (396)	0.718
RaptorX-Roll(358)	0.710
RandomForest(257)	0.708
DNcon(222)	0.701
SVM(81)	0.620

Area under the curve (AUC) for ROC curve calculated using the top L per protein predictions over 96 CASP10 targets. L is the length the protein.

**Table 7:** AUC for the top L/5 predictions on CASP10 targets

Method	AUC
IBGteam [DL] (305)	0.759
RandomForest(257)	0.754
RandomForest (396)	0.748
RaptorX-Roll(358)	0.721
DNcon(222)	0.719
SVM(81)	0.658

Area under the curve (AUC) for ROC curve calculated using the top L/5 per protein predictions over 96 CASP10 targets. L is the length the protein.

### Analysis of DN architecture and feature selection

Given the positive performance of DNcon on the CASP10 benchmark, we decided to extend our analysis of our boosted, deep network architecture to its robustness with respect to the features used and underlying network topology. To assess the sensitivity of the underlying DNs used, we trained and evaluated a number of small, boosted ensembles. First, we studied the effect of the number of layers and nodes per layer. Table 8 shows the performance of 5 different architectures evaluated on the CASP9 dataset. For each architecture considered, a boosted ensemble of 30 DNs was trained using the DNCON_TRAIN dataset and the standard learning procedure outlined in Eickholt and Cheng [17]. The accuracies of the various architectures are very comparable and do not show wide variation even though the number of parameters in the underlying models varies by a wide margin. Thus, in this particular application, the boosted ensemble of DNs is rather robust with respect to the DN's architecture.

**Table 8:** Performance of DN ensembles composed of varying architectures on CASP9 targets

	Acc. Top L/5 (SE)		Acc. Top L (SE)	
	
Architecture	Long	Medium	Long	Medium
500-500-500-350-1	0.174(.012)	0.245(.014)	0.113(.006)	0.144(.008)
750-500-350-1	0.162(.012)	0.231(.013)	0.101(.006)	0.137(.007)
500-500-350-1	0.182(.012)	0.24(.013)	0.122(.006)	0.150(.007)
500-250-1	0.159(.012)	0.243(.131)	0.107(.006)	0.142(.007)
250-250-1	0.169(.010)	0.236(.012)	0.108(.006)	0.142(.007)

To analyze the effect of features used, we divided the input features into five groups based on the type of information they represented or contained. The five groups were residue type (seq), Atchley factors (atch), sequence separation between the residue-residue pair considered (bins), global information consisting of contact potentials, relative positions in sequence and percentage of SS or SA content (globs) and profile information (pssm-ssa). Table 9 lists and describes the feature groups.

**Table 9:** Feature groups used in feature assessment and description

Name	Features included
seq	Residue type (hot encoded)
atch	Atchley factors
bins	The separation in sequence between the residue-residue pair (hot encoded)
globs	Contact potentials, relative position and percentage of helix, loop, beta sheet, exposed
pssm-ssa	Information from the PSSM and predicted secondary structure and solvent accessibility (hot encoded)

Given the time required to train a full, boosted ensemble (approximately 1 day), it was not possible to evaluate all possible combinations of the features groups and therefore we chose to start with only those features directly related to the sequence and then add groups. It is generally accepted that a sequence profile contains more information than the sequence itself and that using a sequence profile often leads to better performance. The drawback is that calculating a sequence profile can be computationally intensive. Thus, the rationale was to see how well a boosted ensemble could perform without profile information and with limited amounts of sequence data. We also evaluated the performance of an ensemble trained on all the feature sets and on a combination of profile information and Atchley factors. Note that all feature groups are used for the ensembles in DNcon.

Table 10 lists the results of our feature set evaluation on the CASP9 dataset. The best performing ensemble does make use of all of the feature groups indicating that all of the features do provide some value. The combination of profile information and Atchley factors is quite effective as well. In general, a great deal of contact information appears to be encoded in the sequence profile since ensembles that included this information outperformed those that did not make use of it.

**Table 10:** Performance of a DN ensemble training on different groups of features on CASP9 targets

Feature set(s)	Acc. Top L/5 (SE)		Acc. Top L (SE)	
	
	Long	Medium	Long	Medium
seq	0.040(.005)	0.042(.004)	0.035(.003)	0.040(.003)
				
seq-atch	0.088(.006)	0.075(.006)	0.077(.005)	0.074(.003)
				
seq-atch-bins	0.078(.005)	0.092(.006)	0.077(.005)	0.085(.005)
				
seq-atch-bins-globals	0.142(.01)	0.202(.007)	0.100(.005)	0.130(.007)
				
seq-atch-bins-globs-pssm-ssa	0.157(.011)	0.221(.013)	0.106(.006)	0.132(.007)
				
pssm-atch	0.168(.012)	0.236(.014)	0.110(.006)	0.130(.007)
				
ALL	0.182(.012)	0.240(.013)	0.122(.006)	0.150(.007)

## Discussion

One outcome of this study is need for broader evaluation metrics for predicted residue-residue contacts. As demonstrated by the strong performance of contact predictors when allowing for less than residue level precision (i.e., evaluating the predictions within a neighbourhood), there is quite a strong contact signal present in the residue-residue contact predictions. Conceptually, this looser definition makes more sense as proteins are dynamic macromolecules. The information we have in experimentally determined structures is simply snapshots of the protein's conformation but minor changes in a protein's shape can and do occur. The noise introduced by such shifts may not make it possible to learn and predict specific residue level contacts from experimentally determined structures. We can, however, learn and predict a number of coarse interactions within the protein chain. This finding explains how predicted residue-residue contacts have been useful in tertiary structure modelling and model quality assessment even though the reported residue-residue accuracies are in the range of 25-35%. Part of the problem has been in properly characterizing the performance. When using a neighbourhood of size 2 (i.e., δ = 2), contact accuracies are in the range of 65-75%. Given that we are able to predict at this level what areas of the protein's chain are in contact, it is not surprising that this information does indeed characterize portions of a protein's conformation.

From a historical perspective, it was quite logical to predict and evaluate contacts at the residue level. These types of predictions naturally translated into inputs that could be used with existing protein reconstruction or modelling pipelines. Given, however, that the contact predictions of state-of-the-art contact predictors are fairly accurate but less precise, what is needed is the development of additional protein reconstruction or modelling pipelines that recognize and exploit this fact. One example of such an approach would be a model evaluation scheme we developed in an earlier work. It allows and considers some slight deviations between predicted contacts and those present in a predicted model [4].

Moving forward, we believe that both residue level (i.e., standard evaluation metrics) and courser level (i.e., by neighbourhood, clustering, etc.) evaluations are important and should be used in assessing the performance of contact predictors. As mentioned, from a historical perspective most attempts to use predicted contact information do so at the residue level and there are undoubtedly situations when residue level accuracy is required. Hence, assessing performance at this level is important but it is also important to better characterize the overall quality and possible value of contact predictions. Knowing that current state-of-the-art contact prediction methods can accurately predict a number of areas of interaction can spur the development of new protein structure prediction techniques that can leverage this information, particularly for the hard modelling targets where additional information is often scarce.

With respect to our residue-residue contact predictor, DNcon, it has shown itself to be rather robust and state-of-the-art approach. In our comparison with other sequence based approaches from CASP10, DNcon consistently performed well and placed in the upper echelon of methods in terms of performance regardless of the evaluation metric used (i.e., residue level accuracy, evaluation by neighbourhood, cluster accuracy and cluster size). The difference between DNcon and other methods was even more pronounced when evaluating hard targets with less than residue level precision. Here, DNcon achieved an accuracy of 0.62 for the top L/10 long range predictions while other approaches reached accuracies in the range of 0.47 to 0.53. In addition to higher accuracy, DNcon's predictions are more dispersed and hence contain more information about the overall conformation of a protein. This is evident by examining the cluster counts in Table 5. DNcon has more clusters present in the top L/10, L/5 and L long range predictions. The interactions represented in these clustered predictions are also more accurately predicted.

The underlying ensembles employed by DNcon showed themselves to be rather robust to the effects of the number of layers or nodes per layer in the DN. This can be seen in Table 8 as the top L/5 medium and long range predictions have accuracies in the ranges of 0.23-0.25 and 0.16-0.18, respectively. While adding additional nodes and layers to the DNs does not appear to increase performance, it also does not negatively affect it, as can often be the case in applications of machine learning.

In analyzing the value of the features sets used, it is clear that the information contained in the PSSM (i.e., sequence profile information) is of great value and significantly contributes to increased performance. For the top L/5 medium and long range contact predictions, it is possible to only use the PSSM and Atchely factors and achieve performances comparable to that of using all features. This is not surprising and the value of profile information in sequence based machine learning methods has been known and used for some time [28]. The drawback to using profile information is that it often comes at a significant computational cost (e.g, running PSI-BLAST for several rounds to create a MSA) and thus makes applications on a genomic scale more difficult. Given the performance of ensembles of DNs which do not use profile information, it is clear that there is a need for further development in this area. In the future, we plan on further investigating and improving the performance of methods which do not require profile information or look for ways in which similar information can be achieved.

## Conclusions

We have presented a study and broad benchmark of DNcon, a method to predict protein residue-residue contacts using deep networks, on the CASP10 dataset. In a comparison with several other sequence based predictors on hard protein modelling targets, DNcon achieved state-of-the-art performance under a variety of evaluation metrics. We also developed and used novel evaluation metrics which characterize a methods performance when allowing for less than residue level precision. In particular, our study shows that state-of-the-art residue-residue contact predictions such as those produced by DNcon do exhibit a strong and distributed contact signal and capable of identifying several areas of interaction in a protein chain. This finding explains how predicted residue-residue contacts have been useful in tertiary structure modelling and model quality assessment even though the reported residue-residue accuracies are in the range of 25-35%. Furthermore, we have demonstrated that the underlying ensembles of DNs used by DNcon are rather robust with respect to architecture and make use of all the features used. DNcon is available as a webservice at http://iris.rnet.missouri.edu/dncon/.

## Competing interests

The authors declare that they have no competing interests.

## Authors' contributions

JE implemented the algorithms and carried out the experiments. JE and JC analyzed the data, wrote and edited the manuscript and approved it.

## Declarations

Publication of this article was supported by NIGMS grant R01GM093123.

This article has been published as part of *BMC Bioinformatics *Volume 14 Supplement 14, 2013: Proceedings of the Tenth Annual MCBIOS Conference. Discovery in a sea of data. The full contents of the supplement are available online at http://www.biomedcentral.com/bmcbioinformatics/supplements/14/S14.
